# The rectus sling to prevent loop colostomy retraction: a case series

**DOI:** 10.1186/1477-7800-2-22

**Published:** 2005-10-20

**Authors:** Gary Atkin, Mike A Scott, Pawan Mathur, Ian C Mitchell

**Affiliations:** 1 Department of Gastrointestinal Surgery, Barnet General Hospital, Wellhouse Lane, Barnet, Herts, EN4 3DJ, UK; 2 70 Rosebery Rd, Muswell Hill, London, N10 2LA, UK

## Abstract

Diverting stomas are being used increasingly in the management of rectal cancer, particularly with low anterior resection following neoadjuvant therapy. We describe a simple anchorage method for loop colostomy using a rectus fascial sling. This has been used successfully in fifteen patients with no complications or evidence of significant spill over of faecal contents into the efferent loop.

## Background

Defunctioning loop colostomy is commonly used in the management of rectal cancer, severe anorectal trauma and perianal sepsis [1]. Traditionally, supporting rods have been used with loop stomas to prevent retraction until maturation occurs. However, these are associated with infection and difficulty applying the stomal appliance [1,2]. We describe a simple technique that obviates the use of the rod whilst still affording secure anchorage of the stoma.

## Method

**Figure 1:**
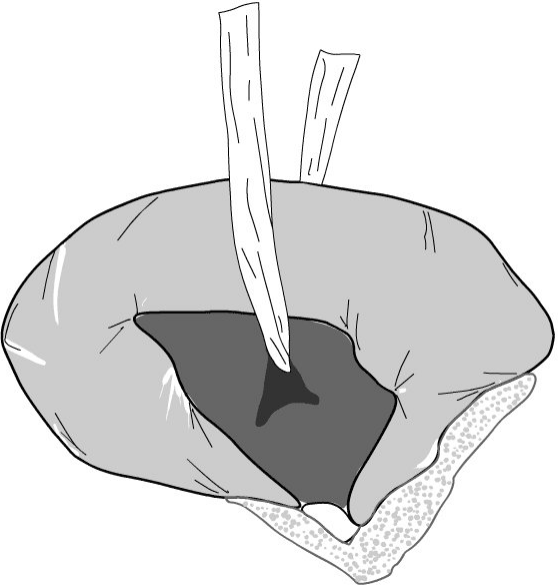
A window in the mesentery is created, and a sling is passed through to aid bowel retraction.

**Figure 2:**
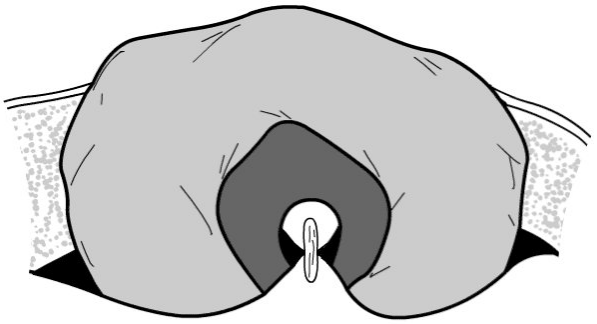
Fascia of rectus sheath is loosely reconstituted under bowel loop through mesenteric window.

Preoperative marking of the stoma site in the left iliac fossa was performed for all patients by a stoma therapist. Patients are positioned supine on the operating table, and a loop colostomy is raised in the standard fashion [3] under general anaesthesia utilising a cruciate incision in the rectus sheath. A suitable loop of sigmoid colon is chosen, and gentle traction is placed on the proximal colon ensuring it is pulled up against the peritoneum. A mesenteric window is created (Figure 1) and the anterior rectus sheath is loosely reconstituted under the colon by suturing the incised corners back together through the mesenteric defect with synthetic polyglactin sutures (Figure 2). The colon is then opened with a transverse incision and the stoma is completed with several mucocutaneous absorbable sutures. The resulting stoma is suitable for use with standard stoma appliances.

## Results

We have used the technique during the elective management of 15 patients with obstructing rectal cancer. There were no peri-operative complications and no evidence of stomal retraction at six months follow up. Of note, we have not encountered problems with 'spill over' of faecal contents into the efferent loop.

## Conclusion

With the increasing use of sphincter sparing procedures for low rectal cancers and the introduction of neoadjuvant therapeutic techniques, there is a greater need for diverting stomas in the surgical management of large intestinal neoplastic disease. Standard technique involves the use of a plastic or glass rod, although rodless stomas have been described [4], and recently a cutaneous suture bridge for loop ileostomy was reported [5]. To the author's knowledge, a rectus sling technique has not been reported previously. By fixing the stoma at the level of the rectus sheath and not the skin, as occurs with cutaneous bridges and supporting rods, the tension on the colonic wall is reduced, thereby minimising the potential risks of retraction and pressure necrosis [2]. Rodless techniques also allow simpler application of stomal appliances, which avoids leakage and increases patient confidence.

Many authors recommend the use of a loop ileostomy when defunctioning the gastrointestinal tract [6]. Loop colostomy still has many advocates, as it is associated with a lower rate of intestinal obstruction and prolonged ileus following anterior resection [7]. It is the authors' practice to use a loop colostomy on a selective basis. The described rectus sling technique for loop colostomy is particularly useful in obese patients, when the distance between the skin and the abdominal cavity is increased, leading to greater tension within the colonic wall and increasing the risk of stomal retraction. In addition, as the colon is anchored securely to the rectus sheath, the authors feel the likelihood of parastomal herniation is reduced, although this would need to be confirmed in a subsequent randomised controlled trial.

## Competing interests

The author(s) declare that they have no competing interests.

## Authors' contributions

GA and MAS prepared the manuscript, whilst PM and IC M provided the data and critically reviewed the article.
